# Dietary Fish and Long-Chain *n*-3 Polyunsaturated Fatty Acids Intake and Risk of Atrial Fibrillation: A Meta-Analysis

**DOI:** 10.3390/nu9090955

**Published:** 2017-08-29

**Authors:** Fu-Rong Li, Guo-Chong Chen, Jiabi Qin, Xianbo Wu

**Affiliations:** 1 Department of Epidemiology, School of Public Health, Southern Medical University, Guangzhou 510000, Guangdong, China; furonglee@gmail.com; 2 Department of Nutrition and Food Hygiene, School of Public Health, Soochow University, Suzhou 215000, Jiangsu, China; guochong_chen@foxmail.com; 3 Saw Swee Hock School of Public Health, National University of Singapore, Singapore 546080, Singapore; 4 Department of Epidemiology and Health Statistics, Xiangya School of Public Health, Central South University, 110 Xiangya Road, Changsha 410078, Hunan, China; qinjiabi123@163.com

**Keywords:** fish, *n*-3 polyunsaturated fatty acids, atrial fibrillation, meta-analysis

## Abstract

Findings on the association between long-term intake of fish or long-chain *n*-3 polyunsaturated fatty acids (PUFAs) and risk of atrial fibrillation (AF) are inconsistent in observational studies. We conducted a meta-analysis of prospective studies to separately examine the associations between fish consumption and dietary intake of *n*-3 PUFAs with the risk of AF. A systematic search was conducted in PubMed and Embase to identify relevant studies. Risk estimates were combined using a random-effect model. Seven prospective cohort studies covering 206,811 participants and 12,913 AF cases were eligible. The summary relative risk of AF for the highest vs. lowest category of fish consumption and dietary intake of *n*-3 PUFAs was 1.01 (95% confidence interval: 0.94–1.09) and 1.03 (95% confidence interval: 0.97–1.09), respectively. These null associations persisted in subgroup and dose-response analyses. There was little evidence of publication bias. This meta-analysis suggests that neither long-term intake of fish, nor of *n*-3 PUFAs were significantly associated with lower risk of AF.

## 1. Introduction

Atrial fibrillation (AF) is one of the major threats to cardiovascular health with a lifetime risk of about 25% [1,2] and a prevalence ranging from 5% to 15% among the elderly aged more than 80 [3]. This common cardiac disorder is estimated to result in more than 1% of Europe’s health-care expenditure because of the subsequent mortality and morbidity. With the growing aging population as well as accumulation of chronic cardiovascular diseases and related risk factors, AF is projected to rise substantially in terms of both prevalence and incidence [4].

Marine-derived long-chain *n*-3 polyunsaturated fatty acids (PUFAs) is predominantly found in fatty fish and the liver of lean fish. *N*-3 PUFAs have received considerable interests for their potential anti-arrhythmic properties, yet evidence from clinical trials and observational studies has not been entirely conclusive [5,6,7,8]. So far, several meta-analyses of clinical trials have been performed to evaluate the effect of *n*-3 PUFAs supplementation on the prevention of AF and, overall, no substantial benefits have been observed [9,10,11,12,13,14]. However, the trials including mostly high-risk population with short-term and high-dose supplementations may not be able to address the association of long-term dietary intake with AF risk. A 2012 meta-analysis, including both prospective observational studies and clinical trials, found no association between fish/fish oil or *n*-3 PUFAs and risk of AF [15]. Nevertheless, the results of this meta-analysis may be limited by combining crude risk estimates instead of variable-adjusted ones. Moreover, it included cohort studies that consisted of patients with myocardial infarction [16] and combined serum and dietary *n*-3 PUFAs in the analysis, which further complicated the interpretation of their findings. Given the limitations of the previous analyses and several new population-based prospective studies regarding the associations between dietary fish and/or *n*-3 PUFAs and risk of AF emerging thereafter [7,8,17], we conducted this meta-analysis to examine the prospective associations between long-term dietary fish and *n*-3 PUFAs intakes and risk of AF.

## 2. Materials

### 2.1. Search Strategy

This meta-analysis was planned, conducted, and reported according to the guidelines of MOOSE (Meta-analysis of Observational Studies in Epidemiology) [18]. A literature search was performed in PubMed and Embase from inception to 18 May 2017 using the following key words: “atrial fibrillation”, “atrial flutter”, “fish”, “seafood”, “fatty acids”, “long-chain omega-3”, “long-chain *n*-3”, “eicosapentaenoic acid”, “docosahexaenoic acid”, “cohort”, “prospective”, and “follow-up”. Bibliographies of the retrieved full articles were also manually screened to identify any additional studies. According to the Tenth Revision of the International Classification of Diseases (ICD-10), AF was defined as atrial fibrillation or atrial flutter (code I48). The literature search was limited to records published in English.

### 2.2. Inclusion Criteria

Studies were included if they met the following criteria: (a) prospective study design; (b) exposure of interest was fish consumption or dietary *n*-3 PUFAs intake; (c) AF as the outcome of interest; and (d) adjusted risk estimates with corresponding 95% confidence intervals (CI) were reported. The most recent publication was included if one cohort was reported in several publications.

### 2.3. Data Extraction

For each eligible study, we extracted the first author’s name, publication year, country of origin, study name, follow-up duration, number of participants and cases, sex, age of participants, exposure, diseases excluded at baseline, and potential confounding factors that were adjusted for. We evaluated study quality with the Newcastle-Ottawa Quality Assessment Scale [19]. According to the scale, nine stars were assigned to each study based on eight items grouped into three categories (selection, comparability and outcome). We finally obtained a total score that summarized the eight aspects of each study. Two researchers (F-R Li and G-C Chen) independently searched the literature, reviewed potentially eligible publications and extracted information from the included studies, with any discrepancy solved by discussion.

### 2.4. Statistical Methods

We considered relative risk (RR) and 95% CI as the effect size for data synthesis, and hazard ratios (HRs) were considered equivalent to RRs [20,21]. A random-effects model which takes into account both within- and between-study variation (heterogeneity) was used to calculate the summary results [22]. For one study [23] that only reported results for fish subtypes (tuna/other fish and fried fish/fish sandwich), we combined the RRs with the inverse variance weight and then included the pooled RR in the meta-analysis. We performed two types of meta-analyses. Our primary analysis pooled RRs for the highest vs. lowest category of fish consumption and dietary *n*-3 PUFAs intake. Subgroup analyses stratified by characteristics of included studies such as sex, region, range of intake, duration of follow-up, age at baseline, and quality scores were also conducted.

Then, a dose-response analysis was conducted to estimate study-specific slopes (linear trends) from the natural logarithm of the extracted RRs and CIs across categories of exposure according to the method proposed by Greenland and Longnecker [24]. The method requires the amount of consumption and the number of cases and person years, as well as corresponding RRs and 95% CIs across different categories. We further evaluated potential nonlinear dose—response relationship by using the restricted cubic spline models with four knots at fixed percentiles (5%, 35%, 65%, and 95%) of the exposure distribution. Potential nonlinearity was assessed by assuming that the coefficient of the second spline was equal to zero. For the intake that was reported as a range rather than a mean or median, the midpoint of the upper and lower boundary of the category was calculated as the average intake. If the extreme categories were open-ended, we assumed that the boundary had the same width as the adjacent category. Results for fish consumption in the study by Brouwer et al. [25] were reported in weight (g/day), we used 105 g as a serving size to perform mutual transformation between consumption weight and frequency [26,27].

Statistical heterogeneity was assessed by using the Q and *I*^2^ statistics (*p* < 0.10 or *I*^2^ > 50% was deemed an indicator of statistically significant heterogeneity) [28]. Publication bias was evaluated by funnel plots, Egger’s test and Begg’s test [29,30]. A two tailed *p* < 0.05 was considered significant unless specifically noted. All analyses were performed by using STATA software (version 12.0; StataCorp LP, College Station, TX, USA).

## 3. Results

### 3.1. Study Characteristics

**Figure 1:**
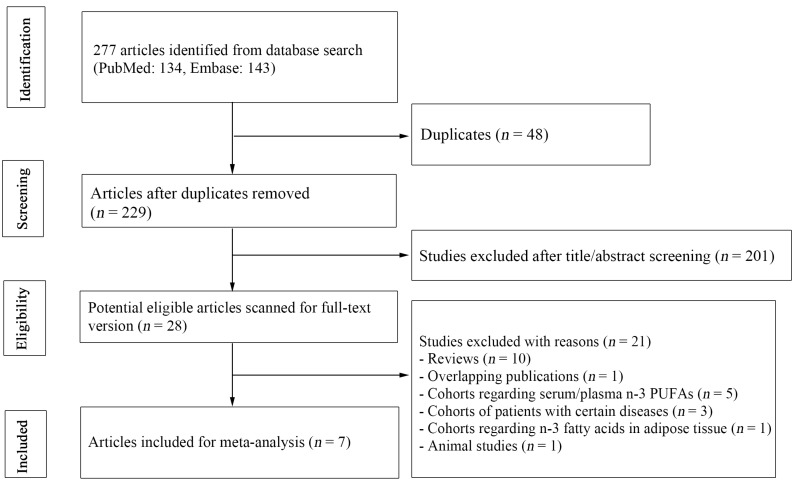
Literature search and study selection.

Figure 1 summarizes the process of literature search. Overall, our database search yielded 277 citations, of which 48 were duplicates between the databases. We further excluded 201 publications after screening titles and abstracts, with 28 potentially relevant articles remained for full-text review. One publication [31] was excluded because it overlapped another more recent one [8] that included the same cohort population. In addition, five publications [32,33,34,35,36] reporting plasma/serum *n*-3 PUFAs were also excluded. Finally, with one publication [17] reporting pooled results of two cohorts, we identified eight prospective studies from seven publications for the meta-analyses [7,8,17,23,25,37,38]. These studies were published between 2004 and 2017, covering 206,811 participants and 12,913 AF cases.

Characteristics of the included studies are summarized in Table 1. The average duration of follow-up ranged between 4 and 17.6 years. Four studies were from the U.S. [7,23,37,38], while the remaining three studies were from Europe [8,17,25]. Most studies recruited both men and women except one that recruited women only [38]. Dietary intakes were measured by food frequency questionnaires in all studies. Study quality assessment showed scores of seven or above (high quality) for all studies, with an average score of 7.6.

### 3.2. Fish Consumption and AF Risk

**Figure 2:**
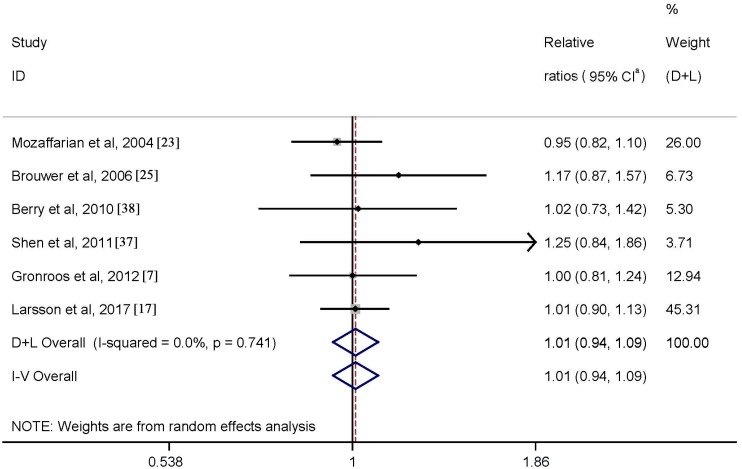
Meta-analysis of fish consumption and AF risk (highest vs. lowest). ^a^ CI: confidence intervals.

One study [8], on fish consumption, was not included because the effect sizes comparing the highest with lowest categories were not provided. Six studies were available for the meta-analysis of the highest vs. lowest fish consumption. Results indicated that high fish intake was not significantly associated with risk of AF (RR = 1.01; 95% CI: 0.94–1.09) with no heterogeneity (*p* = 0.74, *I*^2^ = 0.0%) (Figure 2).

We repeated the analyses by excluding the study by Berry et al. [38], for which total fish consumption was estimated from nonfried fish consumption among women. The combined results remained virtually unchanged (RR = 1.01; 95% CI: 0.93–1.09), without heterogeneity (*p* = 0.60, *I*^2^ = 0.0%). Quantitative assessment (both *p* values for Egger and Begg’s tests ≥0.1) and graphical inspection (not shown) did not indicate evidence of publication bias. The observed null association persisted in the subgroup analyses carried out according to various characteristics of the included studies (Table 2).

### 3.3. N-3 PUFAs Intake and AF Risk

**Figure 3:**
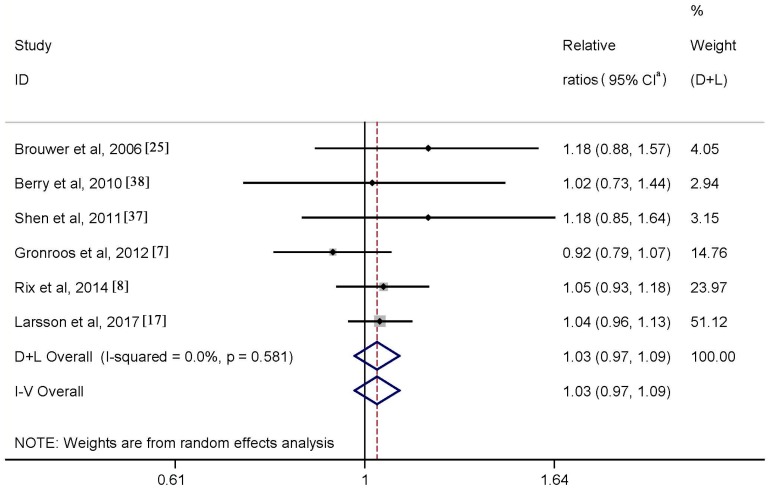
Meta-analysis of *n*-3 PUFAs intake and AF risk (highest vs. lowest). ^a^ CI: confidence intervals.

Data regarding the association between dietary intake of *n*-3 PUFAs and risk of AF were available from six studies. Results of meta-analysis showed a RR of 1.03 (95% CI: 0.97–1.09) when comparing the highest with lowest category of the intake (Figure 3), with no heterogeneity (*p* = 0.581, *I*^2^ = 0.0%). Neither Begg’s test nor Egger’s test revealed publication bias (both *p* values ≥0.6). Funnel plot also suggested absence of publication bias (not shown). Results of subgroup analysis supported the observed null association (Table 3).

### 3.4. Dose-Response Analysis

**Figure 4:**
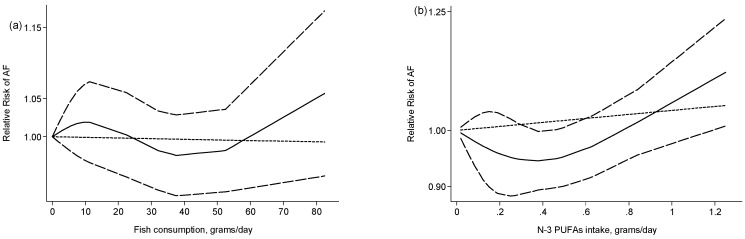
Restricted cubic spline random-effects meta-analysis evaluating potential nonlinear relationships between fish consumption (**a**) and *n*-3 PUFAs intake (**b**) and atrial fibrillation risk. The solid, dashed and dotted lines represent relative risk, 95% confidence interval and estimated linear trends, respectively.

Six studies were combined to explore dose-response relationship between fish consumption and AF risk. The summary RR was 0.99 (95% CI: 0.96–1.02) for each one serving/week increment in fish intake, with low heterogeneity (*p* = 0.261, *I*^2^ = 23.0%). Test for nonlinearity was not significant (*P*_nonlinearity_ = 0.41, Figure 4a).

For the dose-response analysis of *n*-3 PUFAs, one study was excluded because the intake values for each category were not provided [25]. Pooling the remaining five studies suggested a marginally significant association between *n*-3 PUFAs intake and AF, with a summary RR of 1.02 (95% CI: 1.00–1.05) for each 0.3g/day increment in the intake of *n*-3 PUFAs, without heterogeneity (*p* = 0.912, *I*^2^ = 0.0%).There was evidence of a nonlinear association (*P*_nonlinearity_ = 0.006) (Figure 4b). However, this observation should be treated with caution because all data for high intakes were from two studies [8,17].

## 4. Discussion

In this meta-analysis of eight prospective studies with up to 12,000 AF cases occurring among more than 200,000 participants, we found no association between higher fish consumption or *n*-3 PUFAs intake and the development of AF. Such null associations persisted in subgroup analyses based on various study and population characteristics. We observed little evidence of between-study heterogeneity or publication bias. There was some evidence that very high *n*-3 PUFAs intake was associated with a higher risk of AF in the dose-response analysis. However, this observation was sensitive to individual studies and should be treated with great caution.

This meta-analysis has several strengths. First, results of sensitivity analysis and subgroup analysis are consistent, indicating the robustness of our work. Second, the prospective design of included studies provides a more conclusive relationship between dietary intake and AF and eliminates the possibility of recall and selection biases. Third, the dose-response analysis in our study can provide a more direct exhibition of the relationship.

Meanwhile, several limitations should be taken into consideration. Although publication bias was not detected, we still could not rule out such bias because the number of included studies was limited. Also, our findings are based on observational studies and we could not identify causality, but only associations because methodological issues such as the impacts of residual confounding on our results cannot be ruled out. Furthermore, only two of the included studies [8,17] distinguished between lean and fatty fish, whereas the remaining studies reported other subtypes of fish such as dark fish, tuna fish, fried fish, etc. Therefore, we were unable to evaluate whether the association with AF differed by type of fish due to limited data available. In addition, intakes of fish and dietary PUFAs in the highest categories varied widely among studies included, which may affect the interpretation of our results generated from the highest compared with lowest analyses. What’s more, self-reported dietary intake could have led to misclassification of fish and *n*-3 PUFAs intakes, which would likely be non-differential and attenuate a week association to be null. Last but not least, our meta-analysis is unable to specifically investigate the association of dietary supplements of fish oil with AF. However, most of the included studies [8,17,23,37] reported that exclusion of participants taking fish oil supplements did not materially change the results. A study by Larsson et al. [17] also examined the association between fish oil supplements and risk of AF but no significant association was found.

Fish is the major source of *n*-3 PUFAs, and fish-derived *n*-3 PUFAs (docosahexaenoic acid and eicosapentaenoic acid) are reported to be effective on improving blood lipid profile, vascular relaxation, and plaque stability, and thereby may have anti-arrhythmic, anti-inflammatory, and anti-thrombotic properties [39,40,41,42]. Several meta-analyses of observational studies have shown that fish and *n*-3 PUFAs intake are inversely associated with various cardiovascular diseases such as stroke [43], coronary heart disease [27,44], and acute coronary syndrome [45]. However, our work failed to show a significant inverse association between fish consumption and AF. Some factors associated with both fish intake and risk of AF may contribute to this null observation. One of the possibilities is that certain preparation methods may influence the nutrient composition of fish. For example, frying can increase detrimental contents including trans-fatty acid sand oxidation products [46], and thereby diminish the benefits of fish and *n*-3 PUFAs. Higher fish consumption may also accompany higher levels of exposure to harmful substances such as polychlorinated biphenyls, methylmercury, and mercury. These substances may cause damage to the cardiovascular system, and thus counterbalance the effect of other protective factors [47,48]. Further, silent AF is common [4] and people with higher fish intake may represent healthier lifestyles (and therefore better health consciousness) and follow a better systematic electrocardiogram monitoring to avoid a missing diagnosis.

*N*-3 PUFAs play a beneficial role in the cardiac electric activity, and moderate intake of *n*-3 PUFAs may exert beneficial effects on the development of AF through various biological mechanisms [41,42]. Nevertheless, translation of anti-arrhythmic effect of *n*-3 PUFAs into clinical benefits has not yet been proven. Several meta-analyses of clinical trials have investigated the potential health benefits of *n*-3 PUFAs on AF recurrence or postoperative AF, but the findings have not been encouraging [9,10,13,14]. Our results are in line with findings from these meta-analyses based on clinical evidence. However, compared with these meta-analyses, our meta-analysis, though based on observational studies, could also significantly add to the scientific issues we are addressing. We included prospective cohort studies carried out among a large number of generally healthy population with a long duration of follow-up, while clinical trials usually recruit limited number of subjects with specific clinical condition, supplement with a high dose, and have relative shorter duration. The former have the ability to explore an accumulative effect of long-term, low-dose dietary exposure on the primary prevention of a chronic disease among general population. Moreover, observational studies that usually examine results by categorizing the main variable into several levels also have the ability to detect any threshold effect of a dietary exposure that may not be observed in clinical trials, for which a single high-dose supplementation is generally used.

## 5. Conclusions

This meta-analysis suggests no association of fish consumption or *n*-3 PUFAs intake with AF. Additional prospective studies including population with high intake of *n*-3 PUFAs are needed to further explore whether a high-level dietary exposure is detrimental for AF.

## Figures and Tables

**Table 1:** Characteristics of included prospective studies that examined the associations of fish consumption and dietary *n*-3 polyunsaturatedfatty acids (PUFAs) intake with AF risk.

Study, Country	Name	Duration (year)	Participants	No. of Cases	Age (year)	Exposure	Baseline Diseases Excluded	Adjustment for Confounding Factors	Score
Mozaffarian et al., 2004, USA [23]	CHS	12	4815 men and women	980	65–100	Fish	AF	Age, BMI, diastolic blood pressure, education, exercise levels, race, sex, SBP, left ventricular systolic function at baseline, C-reactive protein, smoking, CHD, DM, hypertension, valvular heart disease, energy intake, intake of tuna/other fish, fried fish/fish sandwich, alcohol, beef/pork, cereal fiber, fruits, SFAs, and vegetables	8
Gronroos et al., 2012, USA [7]	ARIC	17.6	14,222 men and women	1604	45–64	Fish and *n*-3 PUFAs	AF	Age, BMI, center, education, exercise levels, race, sex, SBP, HDL and LDL cholesterol levels, smoking, cholesterol treatment, CHD, DM, hypertension, left ventricular hypertrophy, energy intake, and alcohol use	8
Brouwer et al., 2006, Netherland [25]	RS	6.4	2105 men and 3079 women	312	67.4	Fish and *n*-3 PUFAs	AF	Age, sex, HDL and total cholesterol levels, SBP, smoking, DM, MI, energy intake, intake of alcohol, and SFAs	7
Berry et al., 2010, USA [38]	WHI	6	44,720 women	378	50–79	Fish and *n*-3 PUFAs	AF	Age, BMI, education, race, SBP, smoking, DM, hypertension, cardiovascular disease, energy intake, intake of alcohol, fruit, fiber, and vegetable	7
Rix et al., 2014, Denmark [8]	DCHCS	13.6	55,246 men and women	3284	50–64	Fish and *n*-3 PUFAs	Cancer, AF and AFL	Age, BMI, education, sex, SBP, total serum cholesterol, waist circumference, smoking, angina pectoris, DM, hypertension, heart failure, MI, hypercholesterolaemia and/or cholesterol treatment, energy intake, intake of alcohol, fruits, vegetables, red meat, poultry, and fatty dairy products	8
Shen et al., 2011, USA [37]	FHS	4	4231 men and 5409 women	296	62	Fish and *n*-3 PUFAs	AF	Age, BMI, sex, SBP, electrocardiographic PR interval, hypertension, heart failure, and significant heart murmur	7
Larsson et al., 2017, Sweden [17]	COSM, SMC	12	38,960 men and 34,024 women	6059	45–83	Fish and *n*-3 PUFAs	Cancer, AF, ischemic heart disease and heart failure	Age, BMI, education, exercise levels, sex, smoking, DM, family history of MI, hypertension, energy intake, and alcohol use	8

AF, atrial fibrillation; AFL, atrial flutter; ARIC, Atherosclerosis Risk in Communities; BMI, body mass index; CHS, cardiovascular health study; COSM, Cohort of Swedish Men; CHD, coronary heart disease; DM, diabetes mellitus; DCHCS, Diet, Cancer, and Health Cohort Study; FHS, Framingham Heart Study; HDL, high density lipoprotein; LDL, low density lipoprotein; MI, myocardial infarction; RS, Rotterdam Study; SBP, systolic blood pressure; SFAs, saturated fatty acids; SMC, Swedish Mammography Cohort; WHI, Women’s Health Initiative.

**Table 2:** Subgroup analysis for the association of fish consumption and AF risk (highest vs. lowest).

Subgroup	*n*	RR ^a^ (95% CI ^b^)	*P*_heterogeneity_	*I*^2^
Sex				
Male	0	-	-	-
Female	1	1.02 (0.94, 1.09)	-	-
Both	5	1.01 (0.93, 1.09)	0.604	0.0%
Region				
North America	4	0.99 (0.89, 1.11)	0.645	0.0%
Europe	2	1.03 (0.93, 1.14)	0.362	0.0%
Range of intake				
≥3 servings/week	3	1.00 (0.91, 1.09)	0.426	0.0%
<3 servings/week	3	1.05 (0.90, 1.22)	0.689	0.0%
Duration				
≥10 years	3	0.99 (0.91, 1.08)	0.806	0.0%
<10 years	3	1.13 (0.94, 1.38)	0.718	0.0%
Mean/median age at baseline				
≥60 years	5	1.01 (0.93, 1.10)	0.606	0.0%
<60 years	1	1.00 (0.81, 1.24)	-	-
Quality scores				
≥8	3	0.99 (0.91, 1.08)	0.806	0.0%
<8	3	1.13 (0.94, 1.38)	0.718	0.0%

^a^ RR: relative risk; ^b^ CI: confidence intervals.

**Table 3:** Subgroup analysis for the association of *n*-3 PUFAs intake and AF risk (highest vs. lowest).

Subgroup	*n*	RR ^a^ (95% CI ^b^)	*P*_heterogeneity_	*I*^2^
Sex				
Male	0	-	-	-
Female	1	1.02 (0.73, 1.44)	-	-
Both	5	1.03 (0.97, 1.10)	0.437	0.0%
Region				
North America	3	0.97 (0.85, 1.10)	0.383	0.0%
Europe	3	1.05 (0.98, 1.12)	0.713	0.0%
Range of intake				
≥0.3 mg/day	3	1.05 (0.98, 1.12)	0.765	0.0%
<0.3 mg/day	2	1.11 (0.89, 1.38)	0.522	0.0%
Duration				
≥10 years	3	1.02 (0.95, 1.09)	0.330	9.9%
<10 years	3	1.13 (0.94, 1.36)	0.778	0.0%
Mean/median age at baseline				
≥60 years	4	1.05 (0.98, 1.14)	0.758	0.0%
<60 years	2	0.99 (0.87, 1.13)	0.179	44.6%
Quality scores				
≥8	3	1.02 (0.95, 1.09)	0.330	9.9%
<8	3	1.13 (0.94, 1.36)	0.778	0.0%

^a^ RR: relative risk; ^b^ CI: confidence intervals.

## References

[1] Lloyd-Jones D.M., Wang T.J., Leip E.P., Larson M.G., Levy D., Vasan R.S., D’Agostino R.B., Massaro J.M., Beiser A., Wolf P.A. (2004). Lifetime risk for development of atrial fibrillation: The Framingham Heart Study. Circulation.

[2] Mandalenakis Z., Von Koch L., Eriksson H., Dellborg M., Caidahl K., Welin L., Rosengren A., Hansson P.O. (2015). The risk of atrial fibrillation in the general male population: A lifetime follow-up of 50-year-old men. EP Eur..

[3] Camm A.J., Kirchhof P., Lip G.Y., Schotten U., Savelieva I., Ernst S., Van Gelder I.C., Al-Attar N., Hindricks G., Prendergast B. (2010). Guidelines for the management of atrial fibrillation: The Task Force for the Management of Atrial Fibrillation of the European Society of Cardiology (ESC). Eur. Heart J..

[4] Kirchhof P. (2017). The future of atrial fibrillation management: Integrated care and stratified therapy. Lancet.

[5] Aleksova A., Masson S., Maggioni A.P., Lucci D., Fabbri G., Beretta L., Mos L., Paino A.M., Nicolosi G.L., Marchioli R. (2013). *n*-3 polyunsaturated fatty acids and atrial fibrillation in patients with chronic heart failure: The GISSI-HF trial. Eur. J. Heart Fail..

[6] Sorice M., Tritto F.P., Sordelli C., Gregorio R., Piazza L. (2011). *N*-3 polyunsaturated fatty acids reduces post-operative atrial fibrillation incidence in patients undergoing “on-pump” coronary artery bypass graft surgery. Monaldi Arch. Chest Dis..

[7] Gronroos N.N., Chamberlain A.M., Folsom A.R., Soliman E.Z., Agarwal S.K., Nettleton J.A., Alonso A. (2012). Fish, fish-derived *n*-3 fatty acids, and risk of incident atrial fibrillation in the Atherosclerosis Risk in Communities (ARIC) study. PLoS ONE.

[8] Rix T.A., Joensen A.M., Riahi S., Lundbye-Christensen S., Tjonneland A., Schmidt E.B., Overvad K. (2014). A U-shaped association between consumption of marine *n*-3 fatty acids and development of atrial fibrillation/atrial flutter—A Danish cohort study. Europace.

[9] Xin W., Wei W., Lin Z., Zhang X., Yang H., Zhang T., Li B., Mi S. (2013). Fish oil and atrial fibrillation after cardiac surgery: A meta-analysis of randomized controlled trials. PLoS ONE.

[10] Benedetto U., Angeloni E., Melina G., Danesi T.H., Di Bartolomeo R., Lechiancole A., Refice S., Roscitano A., Comito C., Sinatra R. (2013). *n*-3 Polyunsaturated fatty acids for the prevention of postoperative atrial fibrillation a meta-analysis. J. Cardiovasc. Med..

[11] Costanzo S., di Niro V., Di Castelnuovo A., Gianfagna F., Donati M.B., de Gaetano G., Iacoviello L. (2013). Prevention of postoperative atrial fibrillation in open heart surgery patients by preoperative supplementation of *n*-3 polyunsaturated fatty acids: An updated meta-analysis. J. Thorac. Cardiovasc. Surg..

[12] He Z., Yang L., Tian J., Yang K., Wu J., Yao Y. (2013). Efficacy and Safety of Omega-3 Fatty Acids for the Prevention of Atrial Fibrillation: A Meta-analysis. Can. J. Cardiol..

[13] Mariani J., Doval H.C., Nul D., Varini S., Grancelli H., Ferrante D., Tognoni G., Macchia A. (2012). *N*-3 Polyunsaturated Fatty Acids to Prevent Atrial Fibrillation: Updated Systematic Review and Meta-Analysis of Randomized Controlled Trials. J. Am. Heart Assoc..

[14] Zhang B., Zhen Y., Tao A., Bao Z., Zhang G. (2014). Polyunsaturated fatty acids for the prevention of atrial fibrillation after cardiac surgery: An updated meta-analysis of randomized controlled trials. J. Cardiol..

[15] Khawaja O., Gaziano J.M., Djousse L. (2012). A meta-analysis of omega-3 fatty acids and incidence of atrial fibrillation. J. Am. Coll. Nutr..

[16] Macchia A., Monte S., Pellegrini F., Romero M., Ferrante D., Doval H., D’Ettorre A., Maggioni A.P., Tognoni G. (2008). Omega-3 fatty acid supplementation reduces one-year risk of atrial fibrillation in patients hospitalized with myocardial infarction. Eur. J. Clin. Pharmacol..

[17] Larsson S.C., Wolk A. (2017). Fish, long-chain omega-3 polyunsaturated fatty acid intake and incidence of atrial fibrillation: A pooled analysis of two prospective studies. Clin. Nutr..

[18] Stroup D.F., Berlin J.A., Morton S.C., Olkin I., Williamson G.D., Rennie D., Moher D., Becker B.J., Sipe T.A., Thacker S.B. (2000). Meta-analysis of observational studies in epidemiology: A proposal for reporting. Meta-analysis Of Observational Studies in Epidemiology (MOOSE) group. JAMA.

[19] Wells G.A., Shea B., O’Connell D., Peterson J., Welch V., Losos M., Tugwell P. The Newcastle-Ottawa Scale (NOS) for Assessing the Quality of NonrandomizedStudies in Meta-Analyses. http://www.ohri.ca/programs/clinical_epidemiology/oxford.asp.

[20] Pan A., Sun Q., Okereke O.I., Rexrode K.M., Hu F.B. (2011). Depression and risk of stroke morbidity and mortality: A meta-analysis and systematic review. JAMA.

[21] Aune D., Chan D.S., Lau R., Vieira R., Greenwood D.C., Kampman E., Norat T. (2011). Dietary fibre, whole grains, and risk of colorectal cancer: Systematic review and dose-response meta-analysis of prospective studies. BMJ.

[22] DerSimonian R., Laird N. (1986). Meta-analysis in clinical trials. Control Clin. Trials.

[23] Mozaffarian D. (2004). Fish Intake and Risk of Incident Atrial Fibrillation. Circulation.

[24] Greenland S., Longnecker M.P. (1992). Methods for trend estimation from summarized dose-response data, with applications to meta-analysis. Am. J. Epidemiol..

[25] Brouwer I.A., Heeringa J., Geleijnse J.M., Zock P.L., Witteman J.C.M. (2006). Intake of very long-chain *n*-3 fatty acids from fish and incidence of atrial fibrillation. The Rotterdam Study. Am. Heart J..

[26] Zhang Y., Chen J., Qiu J., Li Y., Wang J., Jiao J. (2016). Intakes of fish and polyunsaturated fatty acids and mild-to-severe cognitive impairment risks: A dose-response meta-analysis of 21 cohort studies. Am. J. Clin. Nutr..

[27] He K. (2004). Accumulated Evidence on Fish Consumption and Coronary Heart Disease Mortality: A Meta-Analysis of Cohort Studies. Circulation.

[28] Higgins J.P., Thompson S.G. (2002). Quantifying heterogeneity in a meta-analysis. Stat. Med..

[29] Begg C.B., Mazumdar M. (1994). Operating characteristics of a rank correlation test for publication bias. Biometrics.

[30] Egger M., Davey S.G., Schneider M., Minder C. (1997). Bias in meta-analysis detected by a simple, graphical test. BMJ.

[31] Frost L., Vestergaard P. (2005). *n*-3 Fatty acids consumed from fish and risk of atrial fibrillation or flutter: The Danish Diet, Cancer, and Health Study. Am. J. Clin. Nutr..

[32] Fretts A.M., Mozaffarian D., Siscovick D.S., Djousse L., Heckbert S.R., King I.B., McKnight B., Sitlani C., Sacks F.M., Song X. (2014). Plasma phospholipid saturated fatty acids and incident atrial fibrillation: The Cardiovascular Health Study. J. Am. Heart Assoc..

[33] Fretts A.M., Mozaffarian D., Siscovick D.S., Heckbert S.R., McKnight B., King I.B., Rimm E.B., Psaty B.M., Sacks F.M., Song X. (2012). Associations of Plasma Phospholipid and Dietary Alpha Linolenic Acid With Incident Atrial Fibrillation in Older Adults: The Cardiovascular Health Study. J. Am. Heart Assoc..

[34] Khawaja O., Bartz T.M., Ix J.H., Heckbert S.R., Kizer J.R., Zieman S.J., Mukamal K.J., Tracy R.P., Siscovick D.S., Djoussé L. (2012). Plasma free fatty acids and risk of atrial fibrillation (from the Cardiovascular Health Study). Am. J. Cardiol..

[35] Virtanen J.K., Mursu J., Voutilainen S., Tuomainen T.P. (2009). Serum Long-Chain *n*-3 Polyunsaturated Fatty Acids and Risk of Hospital Diagnosis of Atrial Fibrillation in Men. Circulation.

[36] Wu J.H., Lemaitre R.N., King I.B., Song X., Sacks F.M., Rimm E.B., Heckbert S.R., Siscovick D.S., Mozaffarian D. (2012). Association of plasma phospholipid long-chain omega-3 fatty acids with incident atrial fibrillation in older adults: The cardiovascular health study. Circulation.

[37] Shen J., Johnson V.M., Sullivan L.M., Jacques P.F., Magnani J.W., Lubitz S.A., Pandey S., Levy D., Vasan R.S., Quatromoni P.A. (2011). Dietary factors and incident atrial fibrillation: The Framingham Heart Study. Am. J. Clin. Nutr..

[38] Berry J.D., Prineas R.J., van Horn L., Passman R., Larson J., Goldberger J., Snetselaar L., Tinker L., Liu K., Lloyd-Jones D.M. (2010). Dietary Fish Intake and Incident Atrial Fibrillation (from the Women’s Health Initiative). Am. J. Cardiol..

[39] Siscovick D.S., Raghunathan T.E., King I., Weinmann S., Wicklund K.G., Albright J., Bovbjerg V., Arbogast P., Smith H., Kushi L.H. (1995). Dietary intake and cell membrane levels of long-chain *n*-3 polyunsaturated fatty acids and the risk of primary cardiac arrest. JAMA.

[40] Nestel P.J. (1990). Effects of *n*-3 fatty acids on lipid metabolism. Annu. Rev. Nutr..

[41] Kris-Etherton P.M., Harris W.S., Appel L.J. (2003). Fish consumption, fish oil, omega-3 fatty acids, and cardiovascular disease. Arterioscler. Thromb. Vasc. Biol..

[42] Rix T.A., Mortensen L.M., Schmidt E.B. (2012). Fish, Marine *n*-3 Fatty Acids, and Atrial Fibrillation—Experimental Data and Clinical Effects. Front. Physiol..

[43] Larsson S.C., Orsini N. (2011). Fish consumption and the risk of stroke: A dose-response meta-analysis. Stroke.

[44] Zheng J., Huang T., Yu Y., Hu X., Yang B., Li D. (2012). Fish consumption and CHD mortality: An updated meta-analysis of seventeen cohort studies. Public Health Nutr..

[45] Leung Y.S., Stark K.D., Thanassoulis G., Pilote L. (2014). Fish consumption and acute coronary syndrome: A meta-analysis. Am. J. Med..

[46] Williams M.J., Sutherland W.H., McCormick M.P., de Jong S.A., Walker R.J., Wilkins G.T. (1999). Impaired endothelial function following a meal rich in used cooking fat. J. Am. Coll. Cardiol..

[47] Virtanen J.K., Voutilainen S., Rissanen T.H., Mursu J., Tuomainen T.P., Korhonen M.J., Valkonen V.P., Seppanen K., Laukkanen J.A., Salonen J.T. (2005). Mercury, fish oils, and risk of acute coronary events and cardiovascular disease, coronary heart disease, and all-cause mortality in men in eastern Finland. Arterioscler. Thromb. Vasc. Biol..

[48] Kim S.A., Kim K.S., Lee Y.M., Jacobs D.R., Lee D.H. (2015). Associations of organochlorine pesticides and polychlorinated biphenyls with total, cardiovascular, and cancer mortality in elders with differing fat mass. Environ. Res..

